# Exploring causal relationships between inflammatory cytokines and allergic rhinitis, chronic rhinosinusitis, and nasal polyps: a Mendelian randomization study

**DOI:** 10.3389/fimmu.2023.1288517

**Published:** 2023-11-10

**Authors:** Li Li, Yuanding Zhang, Hong Liu, Tianqi Wang, Junxin Li, Xin Wang

**Affiliations:** ^1^ Department of Otolaryngology-Head and Neck Surgery, Lequn Branch, The First Hospital of Jilin University, Changchun, China; ^2^ Department of Rehabilitation Medicine, The Second Hospital of Jilin University, Changchun, China; ^3^ Department of Otolaryngology-Head and Neck Surgery, The First Hospital of Jilin University, Changchun, China

**Keywords:** allergic rhinitis, chronic rhinosinusitis, nasal polyps, inflammatory cytokines, Mendelian randomization, causal analysis

## Abstract

**Objectives:**

Previous research has suggested connections between specific inflammatory cytokines and nasal conditions, including Allergic Rhinitis (AR), Chronic Rhinosinusitis (CRS), and Nasal Polyps (NP). However, a lack of robust research establishing the causal underpinnings of them. This Mendelian Randomization (MR) study aims to evaluate the causal relationships between 41 inflammatory cytokines and the incidence of AR, CRS and NP.

**Methods:**

This study employed a two-sample MR design, harnessing genetic variations derived from publicly accessible genome-wide association studies (GWAS) datasets. AR data was sourced from a GWAS with 25,486 cases and 87,097 controls (identifier: ukb-b-7178). CRS data originated from a GWAS encompassing 1,179 cases and 360,015 controls (identifier: ukb-d-J32). NP data was extracted from a GWAS involving 1,637 cases and 335,562 controls (identifier: ukb-a-541). The data for 41 inflammatory cytokines were obtained from an independent GWAS encompassing 8,293 participants. Inverse variance weighted (IVW), MR Egger regression and Weighted median were used to evaluate the causalities of exposures and outcomes. A range of sensitivity analyses were implemented to assess the robustness of the results.

**Results:**

The results revealed significant associations between elevated circulating levels of MIP-1α (odds ratio, OR: 1.01798, 95% confidence interval, CI: 1.00217–1.03404, p = 0.02570) and TNF-α (OR: 1.01478, 95% CI: 1.00225–1.02746, p = 0.02067) with an augmented risk of AR in the IVW approach. Heightened levels of circulating IL-2 exhibited a positive correlation with an increased susceptibility to NP in the IVW approach (OR: 1.00129, 95% CI: 1.00017–1.00242, p = 0.02434), whereas elevated levels of circulating PDGF-BB demonstrated a decreased risk of NP (OR: 0.99920, 95% CI: 0.99841–0.99999, p = 0.047610). The MR analysis between levels of 41 inflammatory cytokines and the incidence of CRS yielded no positive outcomes.

**Conclusion:**

This investigation proposes a potential causal association between elevated levels of MIP-1α and TNF-α with an elevated risk of AR, as well as an increased risk of NP linked to elevated IL-2 levels. Furthermore, there appears to be a potential association between increased levels of circulating PDGF-BB and a reduced risk of NP.

## 1 Introduction

For the majority of allergic rhinitis (AR) patients, conservative treatments have shown efficacy; however, a subset of individuals remains unresponsive to standardized conservative approaches, progressing to chronic rhinosinusitis (CRS) and nasal polyps (NP). In some cases of CRS and NP, surgical interventions become necessary following the failure of medical management.

Research has revealed elevated levels of IgE, IgA, IgG, IgG4, IL-4, IL-15, IL-8, and IL-6 in the serum of AR patients (1). The close association between Th2 cytokines and the occurrence and development of AR, CRS and NP has been documented (2, 3). The participation of IL-4, IL-5, IL-13, IL-17, INF-γ, TGF-β, and MMPs in the development of NP and CRS has also been identified (4). Despite the wealth of research exploring the correlation between various inflammatory factors and AR, CRS, and NP, randomized controlled trials (RCTs) are challenging to implement due to multiple limitations in clinical settings. Observational experimental approaches are susceptible to biases due to confounding factors and reverse causality relationships, leading to relatively lower credibility. Often, retrospective analyses of patients’ nasal tissue are used to study the correlation between inflammatory factors and these three conditions, lacking high-quality clinical studies to elucidate the causal relationships between these inflammatory factors and disease occurrence.

This study employs a two-sample Mendelian randomization (MR) approach to explore the causal relationships between inflammatory factors and the incidence of AR, CRS and NP. The aim is to provide insights into the development of these three diseases. Mendelian randomization analysis, proposed by Professor Katan in 1986, is a research method applied in epidemiological observations, utilizing genetic variations as instrumental variables (5). This method examines the causal relationships between exposure factors (phenotypes or diseases) and outcome factors (diseases), analogous to random allocation to treatment and control groups in a randomized controlled trial (6). Genetic variations remain unchanged by other factors and are present from birth, thereby effectively reducing the impact of confounding and reverse causality on the analysis.

While existing MR studies have analyzed the relationship between smoking and AR, as well as NP (7, 8), and the correlation between peripheral blood eosinophils and NP risk (9), this study represents the first MR investigation into the correlation between inflammatory factors and these three diseases. Exploring the influence of inflammatory factors on the occurrence of these three diseases could offer valuable insights for the medical management of these conditions.

## 2 Materials and methods

The current MR analysis utilized summary statistics obtained from previously published GWAS. Informed consent and ethical approval were already obtained during the original data collection, thus supplementary ethical approval is not required for this study.

### 2.1 Study design

**Figure 1:**
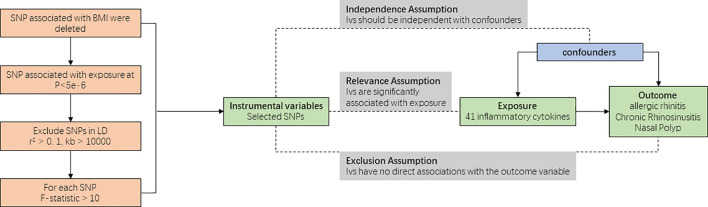
Schematic of the study design in this bidirectional Mendelian randomization (MR) analysis. Significant instrumental variables were selected from 41 inflammatory cytokines with Allergic Rhinitis, Chronic Rhinosinusitis and Nasal Polyp, and the causalities were then explored. Three basic assumptions of MR analysis were illustrated in this causal directed acyclic graph, namely, relevance, independence, and exclusion restrictions.

In the realm of clinical practice, the feasibility of conducting randomized controlled trials (RCTs) can be constrained. Mendelian randomization (MR), a form of instrumental variable (IV) analysis, emerges as a viable alternative, aiming to detect and quantify causal relationships by utilizing genetic variations as instrumental variables. Notably, the chronological sequence is apparent—genetic variations precede the emergence of corresponding traits. This aligns MR with a concept some scholars label as a “natural RCT.” The analytical workflow of the MR analysis in this study is depicted in **Figure 1**.

### 2.2 Data resource

The four datasets employed in this MR analysis were sourced exclusively from publicly available genome-wide association study (GWAS) summary data. These datasets are accessible to the public, and no conflicts of interest are implicated. All datasets pertaining to the exposure groups were procured from the UK Biobank (gwas.mrcieu.ac.uk/datasets). The dataset for AR encompassed 25,486 cases and 87,097 controls of European ancestry. NP data comprised 1,637 cases and 335,562 controls of European ancestry. CRS data encompassed 1,179 cases and 360,015 controls of European ancestry. Data concerning inflammatory cytokines were derived from a study that explored genome variant associations with 41 cytokines and growth factors in 8,293 Finnish individuals (10). This investigation was conducted in Finland, involving the random selection of participants aged between 25 and 74 from five distinct geographic regions. Measurements of cytokine levels were taken from participants’ EDTA plasma, heparin plasma, and serum samples. The analysis exclusively incorporated detectable measurements for each cytokine within the assay range, excluding any cytokines with missing values exceeding 90% (out of 48 cytokines, 7 were excluded). All participants provided written informed consent during data collection. The genetic backgrounds of the four research populations were exclusively of European descent to minimize potential bias resulting from population-specific confounding factors.

### 2.3 Instrumental variable selection

Following a comprehension of the principles underlying Mendelian randomization analysis, instrumental variables were selected based on three fundamental criteria: ①Strong Correlation with Exposure: Instrumental variables were chosen based on their robust correlation with the exposure variable, indicated by a significance threshold of p < 5e-6 and an F-statistic exceeding 10. ②Independence from Confounding Factors: Selected instrumental variables were required to be unrelated to confounding factors that might impact the “exposure-outcome” relationship. ③Unidirectional Influence: Instrumental variables should solely affect the outcome through the exposure variable and not be directly associated with the outcome. Employing these three principles for instrumental variable selection contributes to ensuring the logical coherence of causal relationship analyses.

In the context of chromosomes, certain alleles may exhibit non-random co-occurrence, a phenomenon referred to as linkage disequilibrium (LD). LD often emerges due to the physical proximity of two genes within the genome. For Mendelian randomization analyses, it is crucial to minimize LD between instrumental variables, thereby preserving the randomness of genetic variations, which forms the foundational assumption of Mendelian randomization. This was accomplished by defining thresholds for two parameters, r2 and kb. The chosen thresholds were r2 < 0. 1 and kb < 10000. These values indicate the exclusion of SNPs within a range of 10,000 kb that displayed an r2 value greater than 0.1 in relation to the most significant SNPs.

Given the established association between obesity and inflammation, we conducted a search and removal of SNPs closely correlated with BMI using the Phenoscanner platform (www.phenoscanner.medschl.cam.ac.uk) to mitigate potential interference from body weight on our research. By adhering to these instrumental variable selection criteria, the study endeavors to maintain the validity and reliability of causal inference analyses.

### 2.4 MR analysis

The MR analysis was performed using the “TwoSampleMR” package within R version 4.3.1. Three statistical methods were employed: the inverse variance weighted (IVW) method, the weighted median estimator, and MR-Egger regression. The analysis commenced by assessing associations for each SNP, yielding individual Wald ratios. Subsequently, the inverse variance weighted (IVW) method was applied. This method pooled the Wald ratios using inverse variance weighting, enabling an evaluation of the relationship between the 41 inflammatory cytokines and AR, CRS and NP. It’s noteworthy that the IVW method assumes that all genetic variants are valid instrumental variables. However, this assumption might not always hold practically (11). Acknowledging this assumption’s potential limitations, alternative statistical methods were integrated. These methods do not necessitate the validation of all genetic variants as instrumental variables, ensuring consistent estimates of causal parameters. Among these methods, the weighted median estimator was chosen due to its higher tolerance for invalid instrumental variables. Importantly, this approach can generate credible estimates when more than half of the weight corresponds to valid instrumental variables (12). MR-Egger regression was also introduced. This method offers a valuable complement by relaxing the assumption that all genetic variants function as valid instrumental variables.

### 2.5 Sensitivity analysis

Following the completion of the MR analysis, it becomes crucial to subject the established causal relationships to a stringent quality control protocol. In this regard, various methodologies were employed, encompassing assessments of genetic pleiotropy and heterogeneity, alongside the implementation of the “leave-one-out” technique. This approach aids in preemptively identifying individual SNPs that could potentially exert outsized influence on the resultant outcomes. The MR-Egger approach emerges as a pivotal tool, providing consistent estimations of causative effects under the assumption of Instrument Strength Independent of Direct Effect. This implies that the approach can determine whether significant outcome events can still manifest even in the absence of any influence imparted by exposure. The MR-Egger regression intercept was harnessed as a metric for the horizontal pleiotropy test, wherein a significance threshold of p < 0.05 was deemed indicative of statistical significance (13). To ascertain the presence of heterogeneity within the study, scrutiny was directed toward the Q-pvalue values derived from both IVW analysis and MR-Egger regression. A Q-pvalue exceeding 0.05 suggests the absence of significant heterogeneity. Lastly, the “leave-one-out” test was implemented, entailing the stepwise removal of individual SNPs from the analysis. This process entailed assessing whether the causal effects were notably impacted, ultimately serving as a litmus test for the robustness of the inferred causal relationships.

## 3 Results

### 3.1 Detail information of the included SNPs

The detailed information for the MR analysis of inflammatory factors on AR can be found in **Supplementary Table S1**, utilizing 4 to 18 SNPs as instrumental variables. For the MR analysis of inflammatory factors on NP, refer to **Supplementary Table S2**, which employed 3 to 19 SNPs as instrumental variables. Similarly, detailed information on the MR analysis of inflammatory factors on CRS can be found in **Supplementary Table S3**, utilizing 4 to 20 SNPs as instrumental variables. We used a threshold of p-value less than 0.05 in the IVW analysis and listed all the positive MR analysis results with detailed information on the SNPs used in **Supplementary Tables S4** and **S5**. This comprehensive compilation encompasses a spectrum of particulars, encompassing aspects such as the chromosome location, effect allele (EA), and effect allele frequency (EAF). Notably, the instrumental variables selected achieved F-values exceeding 10. The F statistics play a pivotal role as a reliable gauge of instrumental strength in the context of Mendelian randomization analyses, where values surpassing 10 are widely recognized as indicative of robust instruments (14, 15). Through a systematic selection process, we made concerted efforts to ensure that these SNPs retained a steadfast correlation with the targeted exposure. Simultaneously, we diligently managed their relationships with outcome variables and potential confounding factors to a level deemed acceptable. Consequently, each of these chosen SNPs can confidently be categorized as potent and resilient instrumental variables.

### 3.2 MR results

**Figure 2:**
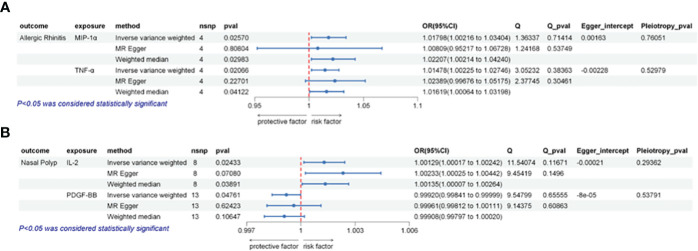
Illustrates the causal relationships between inflammatory factors and the occurrence of AR or NP using three different Mendelian randomization (MR) methods. **(A)** The IVW method indicates that elevated levels of MIP-1α or TNF-α are positively associated with an increased risk of AR (p < 0.05). Sensitivity analysis results demonstrate the absence of horizontal pleiotropy (Pleiotropy_pval > 0.05) or heterogeneity (Q_pval > 0.05) across these analyses. **(B)** The IVW method indicates that elevated levels of IL-2 are positively correlated with an increased risk of NP (p < 0.05), while high levels of PDGF-BB are negatively correlated with NP risk (p < 0.05). Sensitivity analysis results indicate the absence of horizontal pleiotropy (Pleiotropy_pval > 0.05) or heterogeneity (Q_pval > 0.05) in these analyses.

**Figure 3:**
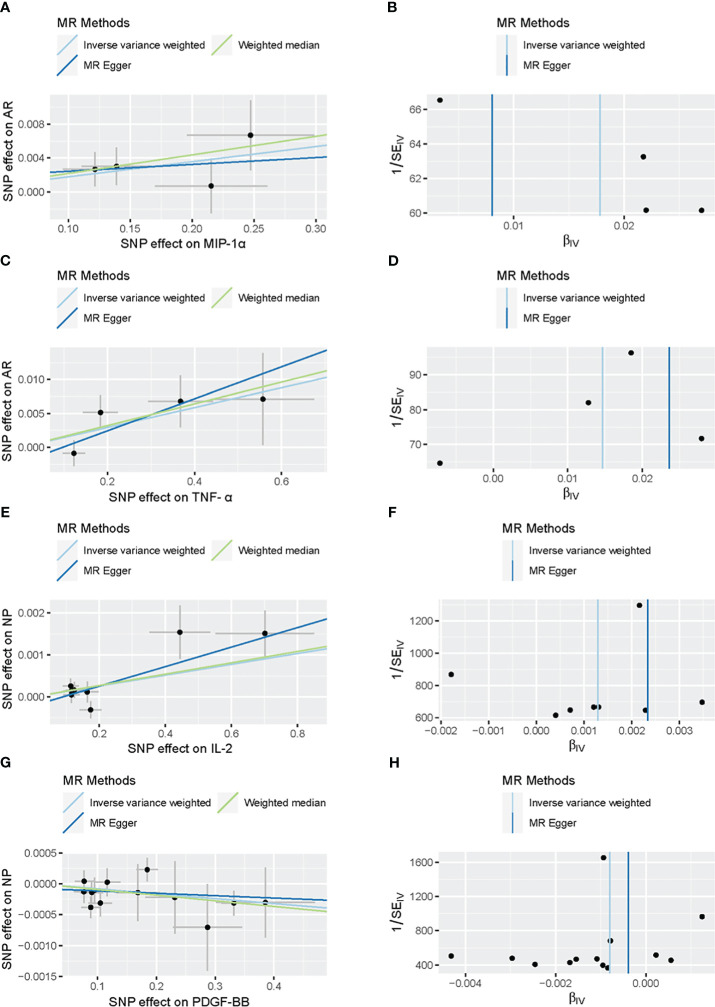
Scatter plots and funnel plots of Mendelian randomization (MR) analyses for MIP-α **(A, B)** and TNF-α **(C, D)** in AR, IL-2 **(E, F)** and PDGF-BB **(G, H)** in Nasal Polyp. Individual inverse variance (IV) associations with cytokine risk are displayed versus individual IV associations with AR in black dots **(A, C, E, G)**. The 95%CI of odd ratio for each IV is shown by vertical and horizontal lines. The slope of the lines represents the estimated causal effect of the MR methods. The funnel plots show the inverse variance weighted MR estimate of each cytokine single-nucleotide polymorphism with AR versus 1/standard error **(B, D, F, H)**.

**Figure 4:**
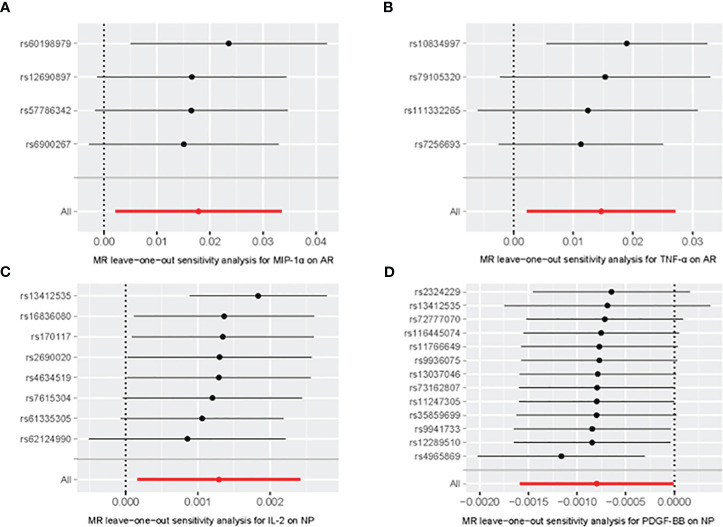
Leave-one-out of SNPs associated between MIP-α **(A)** and TNF-α **(B)** with AR, IL-2 **(C)** and PDGF-BB **(D)** with Nasal Polyp. Each black point represents result of the IVW MR method applied to estimate the causal effect excluding particular SNP from the analysis. Each red point depicts the IVW estimate using all SNPs. No single SNP is strongly driving the overall effect of the overall causal effect in this sensitivity analysis.

Our comprehensive analysis elucidated the causal links between inflammatory cytokines and various nasal conditions. Specifically, we observed a significant correlation between MIP-1α and TNF-α with the incidence risk of AR, as well as a notable association between IL-2 and PDGF-BB with the risk of NP. Importantly, the observed results exhibited no indications of horizontal pleiotropy or heterogeneity. We did not ascertain any association between the 41 inflammatory cytokines and the risk of CRS. The statistically significant outcomes of the MR analysis are visually depicted in **Figure 2**. **Figure 3** provides a graphical representation of the sensitivity analysis conducted within the scope of this study. Additionally, **Figure 4** presents the outcomes yielded by the “leave-one-out” test. Remaining results have been compiled in the **supplementary materials** for comprehensive reference.

### 3.3 Causality between inflammatory cytokines and AR

Our analysis revealed a significant association between heightened genetically predicted circulating MIP-1α levels and an increased risk of AR using the IVW method (OR = 1.01798, 95% CI = 1.00217–1.03404, p = 0.02570, per 1 Standard deviation (SD) increase). Heterogeneity was not detected through Cochran’s Q test (p = 0.71414), and no directional pleiotropy was evident (MR Egger-intercept = -0.00163, p for MR Egger-intercept = 0.76051).

Similarly, the IVW analysis demonstrated a positive correlation between elevated circulating TNF-α levels and an increased risk of AR (OR = 1.01478, 95% CI = 1.00225–1.02746, p = 0.02067, per 1 SD increase). Neither heterogeneity (Cochrane Q test, p = 0.38363) nor directional pleiotropy was identified (MR Egger-intercept = -0.00228, p for MR Egger-intercept = 0.52979).

### 3.4 Causality between inflammatory cytokines and NP

IL-2 exhibited a suggestive link between elevated circulating levels and NP risk in the IVW analysis (OR = 1.00129, 95% CI = 1.00017–1.00242, p = 0.02434, per 1 SD increase). No significant heterogeneity (Cochrane Q test, p = 0.11671) or directional pleiotropy was evident (MR Egger-intercept = -0.00021, p for MR Egger-intercept = 0.29363).

On the other hand, IVW analysis demonstrated a negative correlation between heightened circulating PDGF-BB levels and the risk of NP (OR = 0.99920, 95% CI = 0.99841–0.99999, p = 0.04761, per 1 SD increase). No substantial heterogeneity (Cochrane Q test, p = 0.65555) or directional pleiotropy was identified (MR Egger-intercept = -0.00008, p for MR Egger-intercept = 0.53791).

## 4 Discussion

As presented in the analysis results, the MR analysis indicates that elevated levels of MIP-1α and TNF-α are likely to be correlated with an increased risk of Androgen Receptor (AR). Additionally, elevated IL-2 levels may contribute to an increased risk of Nasal Polyps (NP), whereas higher levels of PDGF-BB might be associated with a reduced risk of NP. Importantly, it was not found that these 41 inflammatory factors are significantly related to the risk of Chronic Rhinosinusitis (CRS). Based on these analysis results and considering existing research on inflammatory factors and these three diseases, several new ideas can be generated.

AR, characterized by chronic inflammation of the nasal mucosa due to allergen exposure, presents with symptoms like sneezing, rhinorrhea, nasal congestion, and irritation. Mediated by Th2 immune responses, it is a type I hypersensitivity reaction where IgE antibodies bind to mast cells upon allergen re-exposure, prompting mast cell degranulation and the release of mediators such as histamine and leukotrienes. Currently, allergen-specific immunotherapy (AIT) has been successfully applied in the treatment of AR. Patients who respond well to AIT can be cured of AR, and even those with partial effectiveness can alleviate the symptoms of AR and, in some cases, allergic asthma (16). However, at the same time, allergy immunotherapy requires a long period, typically around 3 years, to induce immune tolerance in the body, and it comes with a certain risk of desensitization failure. We often use antihistamines with or without intranasal corticosteroids to treat AR. These medications typically only provide symptom relief for AR and cannot cure the condition. Moreover, continuous medication is necessary to maintain the effectiveness of the treatment. This places a regular and frequent medication burden on patients, affecting both their psychological and financial well-being. If left unmanaged, AR can progress to CRS or NP in certain cases. Notably, it’s acknowledged that some cases of CRS or NP may arise without a preceding history of AR; factors like anatomical anomalies or fungal infections can also trigger these conditions. Understanding the roles of inflammatory cytokines in the pathogenesis of AR, CRS and NP can enrich our understanding of these disorders and guide the development of more effective therapeutic strategies. Our study demonstrates that elevated levels of MIP-1α and TNF-α are associated with an increased risk of AR, while elevated levels of IL-2 are associated with an elevated risk of NP. Conversely, increased levels of PDGF-BB are linked to a reduced risk of NP. We did not observe any causal relationships between the 41 listed inflammatory cytokines and the occurrence of CRS.

MIP-1α, also known as CCL3, belongs to the CC chemokine family and serves as a cytokine. Various cell types, including lymphocytes, fibroblasts, epithelial cells, as well as resident and recruited monocytes and macrophages, have been shown to secrete CCL3 (17, 18). CCL3 is an effective chemoattractant for lymphocytes and monocytes. It binds to receptors CCR1, CCR4, and CCR5 on T cells, dendritic cells, B cells, and eosinophils, thereby activating these immune cells and participating in inflammatory responses and immune defenses (19). MIP-1α plays a crucial role in several human diseases, including pulmonary disorders, hematological diseases, rheumatic immune disorders, infectious diseases, and tumors (20). Existing research has indicated its relevance in AR (21). After allergen exposure in AR, the nasal epithelial barrier becomes compromised, releasing various chemokines that initiate an inflammatory cascade by attracting and activating more inflammatory cells. Notably, chemokines like CCL20 and CCL3 facilitate the recruitment of dendritic cells into local lymph nodes, where allergen presentation to naive T cells takes place (22). Specifically, the interaction between MIP-1α and its receptor CCR1 plays a pivotal role in inducing mast cell degranulation during the Early Phase Response of AR (23). These findings corroborate with our study’s outcomes, demonstrating a consistent association between elevated MIP-1α levels and an increased risk of AR.

TNF-α is a widely involved cytokine in inflammatory responses and immune regulation, primarily secreted by macrophages but also by various other cell types. It exerts its effects through two main receptors, namely TNFR1 and TNFR2 (24). Initially, TNF was considered a potential treatment drug for cancer, followed by the opposite concept of targeting TNF for inflammatory diseases (25, 26). Animal experiments have demonstrated that knocking out TNF-alpha in AR mouse models can alleviate allergic reactions in mice (27). Some studies have reported that anti-TNF-α antibodies can prevent house dust mite (HDM)-induced allergic airway inflammation (28). In *in vitro* experiments, the nasal cavity’s response to salivarius bacteria was found to stimulate the expression of pro-inflammatory cytokines such as IL-6, IL-8, and TNF-α, as well as epithelial cytokines like IL-33 and TSLP. This cascade triggers Th2 cell-mediated allergic responses and leads to the release of chemoattractant eotaxin-1 (CCL11) (29). Another study combined an anti-TNF-α nanobody (V) with tannic acid (V/TA) as a functional antibody drug candidate and found that it could inhibit cytokine release in an ovalbumin (OVA)-induced AR murine model (30). In our study, the observed association between elevated levels of TNF-α and increased AR risk aligns with previous research findings.

Interleukin-2 (IL-2) is a widely recognized cytokine that holds a significant role in the growth and proliferation of various immune cells Notably, IL-2 has emerged as a crucial immunotherapy cytokine for treating diverse conditions, including cancer. It orchestrates the expansion and differentiation of immune cells such as T cells, NK cells, and B cells. Furthermore, IL-2 stimulates the differentiation of CD4+ and CD8+ T cells into memory cells and terminally differentiated lymphocytes, while also enhancing the cytolytic activity of NK cells and T-lymphocytes (31). In immune response regulation and maintaining immune tolerance, IL-2 plays a pivotal role (32). Research has indicated that peripheral blood IL-2 levels are more frequently detected in patients with CRS accompanied by cystic formation compared to normal patients. The study suggests that CRSwP with cystic formation is marked by the emergence of a T2 type immune response and heightened inflammation-related tissue damage (33). However, other studies have demonstrated a decrease in the expression levels of IL-2, IL-2R, and pSTAT5 in NP compared to normal controls (34). Notably, these contradictory findings could be attributed to differences in specimen collection and the intricate regulatory role of IL-2 within Th2 immune responses, which significantly influence the development of NP (35). Our study underscores a positive association between elevated peripheral levels of IL-2 and increased AR risk, potentially contributing to a better understanding of IL-2’s role in AR.

PDGF-BB, an isoform of platelet-derived growth factor (PDGF), consists of two B chains. This cytokine, known as Platelet-Derived Growth Factor-BB (PDGF-BB), is endowed with mitogenic, differentiating, chemotactic, and angiogenic properties. Its involvement spans wound healing, but as part of the PDGF family, PDGF-BB has been implicated in various pathological conditions (36). In tumor cell conditions, PDGF-BB plays versatile roles, functioning both protectively and pathogenically (37). Indeed, PDGF-BB is considered a pivotal mediator in promoting wound healing and tissue restoration. It orchestrates angiogenesis through stimulating vascular endothelial growth factor (VEGF) secretion and contributes to the maintenance of vascular stability in newly formed vessels (38). The scientific understanding of PDGF-BB’s involvement in the genesis and progression of NP is rather limited. Some researchers have identified eosinophils as synthesizers of PDGF-B in chronic airway inflammations, including nasal polyposis and bronchial asthma (39). Our study unveils an inverse correlation between elevated levels of PDGF-BB and the risk of NP occurrence. Based on the physiological functions of PDGF-BB, it is conceivable that its effects might operate in two keyways. Firstly, it could potentially facilitate the repair of nasal mucosal damage, thereby reducing the formation of polypoid mucosa. Secondly, PDGF-BB may play a role in stabilizing blood vessels within the nasal mucosa. This stabilization could lead to an improvement in local oxygen levels and blood supply. As a result, the accumulation of inflammatory mediators in the nasal cavity could be mitigated, subsequently reducing the likelihood of NP formation.

In this study, we did not find a potential causal relationship between the 41 inflammation factors included and the onset of chronic rhinosinusitis (CRS). Soyka and colleagues (40) discovered that high levels of Type 2 cytokines IL-13 and IL-4 present in eosinophilic polyps might act as inductors for chronic epithelial-to-mesenchymal transition (EMT) in CRS. EMT is considered a significant mechanism in the formation of nasal polyps and sinusitis. Gevaert and his team (41) found that using anti-IL-5 treatment in patients with CRS with nasal polyps (CRSwNP) reduces eosinophilic infiltration, thereby reducing the size of nasal polyps. However, our study did not find an association between these inflammation factors and the onset of CRS. On one hand, it might be due to the heterogeneity of the disease in our study, which included data from both CRSwNP and CRS without nasal polyps (CRSsNP) patients, causing some interference in the analysis of the results. On the other hand, it could be because the sample size was not large enough, leading to the potential causal associations not being demonstrated. Alternatively, it’s possible that the 41 inflammation factors investigated in this study indeed have no causal relationship with the occurrence of CRS, and the absence of this causal link requires rigorous experiments in subsequent studies to confirm.

Certainly, our study does come with certain limitations. Firstly, the interconnected nature of AR, CRS and NP makes their complete differentiation challenging, as each condition exhibits distinct characteristics. Utilizing summarized-level GWAS data in our study precluded subgroup analyses based on population characteristics or disease features, limiting a more detailed exploration of these interrelationships. Furthermore, even with careful SNP selection, our understanding of the relationship between SNPs and traits remains incomplete, which could introduce potential pleiotropy and horizontal heterogeneity, thereby potentially attenuating the power of our causal effect analyses. Secondly, as a method of instrumental variable analysis, Mendelian Randomization (MR) can only offer potential insights into causality but cannot directly prove causal relationships. We still need to combine prospective cohort studies with molecular-level mechanistic research to demonstrate whether this causality indeed exists. Additionally, our study’s focus on individuals of European ancestry raises questions about the generalizability of our findings to other populations. As we know, allergic rhinitis is more common in developed countries, while nasal polyps are more prevalent in Asia. The association of these two conditions with chronic rhinosinusitis (CRS) might lead to differences in the etiological mechanisms and incidence rates of CRS among different ethnic groups. Although there is a lack of corresponding authoritative survey reports, analyzing from the perspective of disease mechanisms, the living environments and allergens encountered differ across regions and ethnicities. Moreover, the genetic backgrounds of different ethnic groups vary significantly. Therefore, whether our conclusion can be applied to populations outside Europe still requires further exploration through future research.

While acknowledging our study’s limitations, its novel methodology and significant findings underscore its contributions to our understanding of the relationships between inflammatory factors and the risks of AR, CRS and NP. Building on this foundation, further research can explore different avenues. On one hand, the focus could be on adjusting the levels of specific inflammatory factors in the peripheral circulation to prevent or treat these three diseases. For example, using monoclonal antibodies to reduce the concentration of certain inflammatory factors or supplementing specific inflammatory factors exogenously could be studied to observe their potential impact on the risk of developing these three diseases. On the other hand, conducting more in-depth molecular-level mechanistic studies regarding the potential causes of this underlying causal association could provide new insights for understanding and treating these three conditions. Such research endeavours hold the potential to open new doors in our understanding and management of allergic rhinitis, chronic rhinosinusitis, and nasal polyps.

## 5 Conclusion

Our findings suggest that elevated levels of MIP-1α and TNF-α are likely to be correlated with an increased risk of AR, while elevated IL-2 levels may contribute to an increased risk of NP. On the other hand, our study suggests that higher levels of PDGF-BB might be associated with a reduced risk of NP. No significant correlations were observed between the levels of these 41 inflammatory-related cytokines and the risk of CRS.

The utilization of Mendelian randomization and large-scale GWAS data provides robust evidence for these associations. Further research is needed to validate and extend these findings. Understanding the roles of these inflammatory factors in the pathogenesis of these diseases could offer new avenues for the development of more effective therapeutic strategies.

## Data availability statement

The original contributions presented in the study are included in the article/**Supplementary Material**. Further inquiries can be directed to the corresponding author.

## Ethics statement

This MR analysis utilized summary statistics obtained from previously published GWAS. Ethical approval were already obtained during the original data collection, thus supplementary ethical approval is not required for this study. The studies were conducted in accordance with the local legislation and institutional requirements. Informed consent were already obtained during the original data collection, thus supplementary ethical approval is not required for this study.

## Author contributions

LL: Formal Analysis, Software, Writing – original draft, Conceptualization, Methodology. YZ: Data curation, Writing – review & editing, Conceptualization, Supervision. HL: Writing – review & editing, Investigation. TW: Visualization, Writing – original draft. JL: Investigation, Project administration, Writing – original draft. XW: Conceptualization, Formal Analysis, Methodology, Supervision, Writing – original draft, Writing – review & editing.

## References

[1] Chai W Zhang X Lin M Chen Z Wang X Wang C. Allergic rhinitis, allergic contact dermatitis and disease comorbidity belong to separate entities with distinct composition of T-cell subsets, cytokines, immunoglobulins and autoantibodies. Allergy Asthma &amp; Clin Immunol (2022) 18(1):1–12. doi: 10.1186/s13223-022-00646-6. 2. PMC8840545 35148790

[2] Min HJ Park JS Kim KS Park SY Choi H Seo JH. Th2 cytokines-DUOX2-ros-HMGB1 translocation axis is important in the pathogenesis of allergic rhinitis. Clin Science. (2021) 135(3):483–94. doi: 10.1042/cs20201212 33458745

[3] Zhang Y Derycke L Holtappels G Wang XD Zhang L Bachert C. Th2 cytokines orchestrate the secretion of MUC 5 AC and MUC 5B in IL-5-positive chronic rhinosinusitis with nasal polyps. Allergy (2019) 74(1):131–40. doi: 10.1111/all.13489 29802623

[4] Schleimer RP. Immunopathogenesis of chronic rhinosinusitis and nasal polyposis. Annu Rev Pathology: Mech Disease. (2017) 12(1):331–57. doi: 10.1146/annurev-pathol-052016-100401 PMC5514544 27959637

[5] Freuer D Linseisen J Meisinger C. Association between inflammatory bowel disease and both psoriasis and psoriatic arthritis. JAMA Dermatol (2022) 158(11):1262. doi: 10.1001/jamadermatol.2022.3682 36103169 PMC9475439

[6] Davies NM Holmes MV Davey Smith G. Reading Mendelian Randomisation Studies: A guide, glossary, and checklist for Clinicians. BMJ (2018) 362. doi: 10.1136/bmj.k601 PMC6041728 30002074

[7] Wang S Qi L Wei H Jiang F Yan A. Smoking behavior might affect allergic rhinitis and vasomotor rhinitis differently: A Mendelian randomization appraisal. World Allergy Organ J (2022) 15(2):100630. doi: 10.1016/j.waojou.2022.100630 35228855 PMC8844647

[8] Zhang Z Li G Yu L Jiang J Li R Zhou S. Causal relationships between potential risk factors and chronic rhinosinusitis: A bidirectional two-sample mendelian randomization study. Eur Arch Oto-Rhino-Laryngology. (2023) 280(6):2785–93. doi: 10.1007/s00405-022-07798-6 PMC9803893 36585990

[9] Huang G-J Chen Z-Q Fan Z-J Li S-H. The causal association between peripheral blood eosinophils and nasal polyps: A Mendelian randomization study. Eur Arch Oto-Rhino-Laryngology. (2023) 280(9):4285–90. doi: 10.1007/s00405-023-08129-z 37466661

[10] Ahola-Olli AV Würtz P Havulinna AS Aalto K Pitkänen N Lehtimäki T. Genome-wide association study identifies 27 loci influencing concentrations of circulating cytokines and growth factors. Am J Hum Genet (2017) 100(1):40–50. doi: 10.1016/j.ajhg.2016.11.007 27989323 PMC5223028

[11] Burgess S Butterworth A Thompson SG. Mendelian randomization analysis with multiple genetic variants using summarized data. Genet Epidemiol (2013) 37(7):658–65. doi: 10.1002/gepi.21758 PMC4377079 24114802

[12] Bowden J Davey Smith G Haycock PC Burgess S. Consistent estimation in Mendelian randomization with some invalid instruments using a weighted median estimator. Genet Epidemiol (2016) 40(4):304–14. doi: 10.1002/gepi.21965 PMC4849733 27061298

[13] Bowden J Davey Smith G Burgess S. Mendelian randomization with invalid instruments: Effect estimation and bias detection through Egger regression. Int J Epidemiol (2015) 44(2):512–25. doi: 10.1093/ije/dyv080 PMC4469799 26050253

[14] Pierce BL Ahsan H VanderWeele TJ. Power and instrument strength requirements for Mendelian randomization studies using multiple genetic variants. Int J Epidemiol (2011) 40(3):740–52. doi: 10.1093/ije/dyq151 PMC3147064 20813862

[15] Palmer TM Lawlor DA Harbord RM Sheehan NA Tobias JH Timpson NJ. Using multiple genetic variants as instrumental variables for modifiable risk factors. Stat Methods Med Res (2012) 21(3):223–42. doi: 10.1177/0962280210394459 PMC3917707 21216802

[16] Ponda P Carr T Rank MA Bousquet J. Nonallergic rhinitis, allergic rhinitis, and immunotherapy: advances in the last decade. J Allergy Clin Immunology: In Pract (2023) 11(1):35–42. doi: 10.1016/j.jaip.2022.09.010 36152989

[17] Danforth JM Strieter RM Kunkel SL Arenberg DA VanOtteren GM Standiford TJ. Macrophage inflammatory protein-1α expression in *vivo* and in *vitro*: The role of lipoteichoic acid. Clin Immunol Immunopathology. (1995) 74(1):77–83. doi: 10.1006/clin.1995.1011 7994929

[18] Lindell DM Standiford TJ Mancuso P Leshen ZJ Huffnagle GB. Macrophage inflammatory protein 1alpha/CCL3 is required for clearance of an acute *Klebsiella pneumoniae* pulmonary infection. Infection Immunity. (2001) 69(10):6364–9. doi: 10.1128/iai.69.10.6364-6369.2001 PMC98771 11553580

[19] Schaller TH Batich KA Suryadevara CM Desai R Sampson JH. Chemokines as adjuvants for immunotherapy: Implications for immune activation with CCL3. Expert Rev Clin Immunol (2017) 13(11):1049–60. doi: 10.1080/1744666x.2017.1384313 PMC6020048 28965431

[20] Nath A Chattopadhya S Chattopadhyay U Sharma NK. Macrophage inflammatory protein (MIP)1α and MIP1β differentially regulate release of inflammatory cytokines and generation of tumoricidal monocytes in Malignancy. Cancer Immunology Immunother (2006) 55(12):1534–41. doi: 10.1007/s00262-006-0149-3 PMC11030200 16518599

[21] Li Z Yu S Jiang Y Fu Y. Chemokines and chemokine receptors in allergic rhinitis: From mediators to potential therapeutic targets. Eur Arch Oto-Rhino-Laryngology. (2022) 279(11):5089–95. doi: 10.1007/s00405-022-07485-6 35732904

[22] Liu C Zhang X Xiang Y Qu X Liu H Liu C. Role of epithelial chemokines in the pathogenesis of airway inflammation in asthma (review). Mol Med Rep (2018) 6935–6941. doi: 10.3892/mmr.2018.8739 29568899

[23] Miyazaki D Nakamura T Toda M Cheung-Chau K-W Richardson RM Ono SJ. Macrophage inflammatory protein–1α as a costimulatory signal for mast cell–mediated immediate hypersensitivity reactions. J Clin Invest (2005) 115(2):434–42. doi: 10.1172/jci18452 PMC544033 15650768

[24] Kalliolias GD Ivashkiv LB. TNF biology, pathogenic mechanisms and emerging therapeutic strategies. Nat Rev Rheumatol (2016) 12(1):49–62. doi: 10.1038/nrrheum.2015.169 26656660 PMC4809675

[25] Feldmann M. Translating molecular insights in autoimmunity into effective therapy. Annu Rev Immunol (2009) 27(1):1–27. doi: 10.1146/annurev-immunol-082708-100732 19007330

[26] Cerami A. The value of failure: The discovery of TNF and its natural inhibitor erythropoietin. J Internal Med (2011) 269(1):8–15. doi: 10.1111/j.1365-2796.2010.02319.x 21158973

[27] Iwasaki M Saito K Takemura M Sekikawa K Fujii H Yamada Y. TNF-α contributes to the development of allergic rhinitis in mice. J Allergy Clin Immunol (2003) 112(1):134–40. doi: 10.1067/mai.2003.1554 12847490

[28] Steelant B Seys SF Van Gerven L Van Woensel M Farré R Wawrzyniak P. Histamine and T helper cytokine–driven epithelial barrier dysfunction in allergic rhinitis. J Allergy Clin Immunol (2018) 141(3):951–63. doi: 10.1016/j.jaci.2017.08.039 29074456

[29] Miao P Jiang Y Jian Y Shi J Liu Y Piewngam P. Exacerbation of allergic rhinitis by the commensal bacterium streptococcus salivarius. Nat Microbiol (2023) 8(2):218–30. doi: 10.1038/s41564-022-01301-x PMC10062442 36635572

[30] Fu S Cao Z Huang B Yin T Huang C Bi Z. Tannic acid assisted anti-TNF-α nanobody assembly modulating the epithelial barrier dysregulation of allergic rhinitis. Nano Res (2023) 16(7):9781–91. doi: 10.1007/s12274-023-5646-6

[31] Dhupkar P Gordon N. Interleukin-2: Old and new approaches to enhance immune-therapeutic efficacy. Adv Exp Med Biol (2017) 995:33–51. doi: 10.1007/978-3-319-53156-4_2 28321811

[32] Malek TR Castro I. Interleukin-2 receptor signaling: At the interface between tolerance and immunity. Immunity. (2010) 33(2):153–65. doi: 10.1016/j.immuni.2010.08.004 PMC2946796 20732639

[33] Kokorina OV Boeva VI Apalko SV Vologzhanin DA Dvoryanchikov VV Shcherbak SG. Cytokine profile of chronic rhinosinusitis without polyps. Vestnik otorinolaringologii. (2022) 87(4):51. doi: 10.17116/otorino20228704151 36107181

[34] Wang X Hu G Kou W Hong S. The role of interleukin-2 pathway in pathogenesis of nasal polyps. Lin Chuang er bi yan hou tou Jing wai ke za zhi= J Clin Otorhinolaryngology Head Neck Surg (2014) 28(8):509–12. doi: 10.13201/j.issn.1001-1781.2014.08.001 25007660

[35] Shen Y Zhang N Yang Y Hong S Bachert C. Local immunoglobulin E in nasal polyps: Role and modulation. Front Immunol (2022) 13:961503. doi: 10.3389/fimmu.2022.961503 36159836 PMC9492990

[36] Alvarez RH Kantarjian HM Cortes JE. Biology of platelet-derived growth factor and its involvement in disease. Mayo Clin Proc (2006) 81(9):1241–57. doi: 10.4065/81.9.1241 16970222

[37] Yi B Williams PJ Niewolna M Wang Y Yoneda T. Tumor-derived platelet-derived growth factor-BB plays a critical role in osteosclerotic bone metastasis in an animal model of human breast cancer. Cancer Res (2002) 62(3):917–23. 11830552

[38] Chen Y Jiang L Lyu K Lu J Long L Wang X. A promising candidate in tendon healing events—PDGF-BB. Biomolecules. (2022) 12(10):1518. doi: 10.3390/biom12101518 36291727 PMC9599567

[39] Ohno I Nitta Y Yamauchi K Hoshi H Honma M Woolley K. Eosinophils as a potential source of platelet-derived growth factor B-Chain (PDGF-B) in nasal polyposis and bronchial asthma. Am J Respir Cell Mol Biol (1995) 13(6):639–47. doi: 10.1165/ajrcmb.13.6.7576701. 1. 7576701

[40] Soyka MB Wawrzyniak P Eiwegger T Holzmann D Treis A Wanke K. Defective epithelial barrier in chronic rhinosinusitis: the regulation of tight junctions by IFN-γ and IL-4. J Allergy Clin Immunol (2012) 130(5):1087–96. doi: 10.1016/j.jaci.2012.05.052 22840853

[41] Gevaert P Van Bruaene N Cattaert T Van Steen K Van Zele T Acke F. Mepolizumab, a humanized anti–IL-5 mAb, as a treatment option for severe nasal polyposis. J Allergy Clin Immunol (2011) 128(5):989–95. doi: 10.1016/j.jaci.2011.07.056 21958585

